# Self-Reported Modifiable Risk Factors of Cardiovascular Disease among Seafarers: A Cross-Sectional Study of Prevalence and Clustering

**DOI:** 10.3390/jpm11060512

**Published:** 2021-06-04

**Authors:** Getu Gamo Sagaro, Gopi Battineni, Marzio Di Canio, Francesco Amenta

**Affiliations:** 1 Telemedicine and Telepharmacy Center, School of Medicinal and Health Products Sciences, University of Camerino, 62032 Camerino, Italy; gopi.battineni@unicam.it (G.B.); mdicanio@cirmservizi.it (M.D.C.); francesco.amenta@unicam.it (F.A.); 2 International Radio Medical Center (C.I.R.M.), Research Department, 00144 Rome, Italy

**Keywords:** seafarers, hypertension, cardiovascular disease, occupation, diabetes, body mass index, cigarette smoking, modifiable risk factors

## Abstract

Background: Cardiovascular diseases (CVD) are the major cause of work-related mortality from diseases onboard ships in seafarers. CVD burden derives mainly from modifiable risk factors. To reduce the risk factors and the burden of CVD onboard ships in seafarers, it is important to understand the up-to-date prevalence of modifiable risk factors. The primary purpose of this study was to assess the prevalence and clustering of self-reported modifiable CVD risk factors among seafarers. We have also explored the association between socio-demographic and occupational characteristics and reported modifiable CVD risk factor clustering. Materials and methods: A cross-sectional study was conducted among seafarers from November to December 2020 on board ships. In total, 8125 seafarers aged 18 to 70 were selected from 400 ships. Data were collected using a standardized and anonymous self-reported questionnaire. The prevalence value for categorical variables and mean differences for continuous variables were compared using chi-square and independent sample t-tests. Multinomial logistic regression models were performed to identify independent predictors for modifiable CVD risk factor clustering. Results: Out of a total of 8125 seafarers aged ≥18 years on selected vessels, 4648 seafarers volunteered to participate in the survey, with a response rate of 57.2%. Out of 4318 participants included in analysis, 44.7% and 55.3% were officers and non-officers, respectively. The prevalence of reported hypertension, diabetes, current smoking and overweight or obesity were 20.8%, 8.5%, 32.5%, and 44.7%, respectively. Overall, 40%, 20.9%, 6% and 1.3% of the study participants respectively had one, two, three and four modifiable CVD risk factors. Older age (51+ years) (odds ratio (OR): 3.92, 95% confidence interval (CI): 2.44–6.29), being non-officers (OR: 1.36, 95% CI: 1.09–1.70), job duration (10–20 years) (OR: 2.73, 95% CI: 2.09–3.57), job duration (21+ years) (OR: 2.60, 95% CI: 1.79–3.78), working 57–70 h per week (OR: 2.03, 95% CI: 1.65–2.49) and working 71+ h per week (OR: 3.08, 95% CI: 2.42–3.92) were independent predictors for at least two self-reported modifiable CVD risk factor clustering. Conclusion: The results of our study demonstrate that more than four in six (68.5%) seafarers aged between 19 and 70 years have at least one of the modifiable CVD risk factors. Therefore, CVD prevention and modifiable risk factors reduction strategies targeting high-risk groups should be designed and implemented on board ships.

## 1. Introduction

Globally, cardiovascular diseases (CVD) are the leading causes of mortality and disability [1]. The prevalence of overall CVD increased from 271 million in 1990 to 523 million in 2019, and mortality due to CVD also increased from 12.1 million to 18.6 million respectively from 1990 to 2019 worldwide [1]. CVD are responsible for approximately 31% (17.9 million) of all death worldwide, and four out of five (80%) deaths from these diseases were due to heart attacks and strokes [2].

CVDs are also the major cause of work-related mortality from diseases among seafarers’ onboard ships. A study conducted on British merchant shipping reported that 4601 (19.8%) of 23,291 work-related deaths from 1919 to 2005 were due to CVD [3]. Another study found that 147 (38.4%) out of the 384 documented deaths at sea were from CVDs [4]. Various studies have reported that a high percentage of deaths among seafarers was due to CVDs in Polish vessels (62 (19%) of 324 deaths) [5], on Danish merchant ships (35 (66%) of 53 deaths) [6], on Singapore ships (45 (65%) of 69 deaths) [7], and in Isle of Man shipping (16 (80%) of 20 deaths) [8]. In terms of morbidity, CVDs are among the top five (gastrointestinal, CV, musculoskeletal, dermatological and respiratory disorders) major cause of illness from diseases at sea among seafarers [9,10,11,12].

CVD burden attributable to modifiable risk factors, namely hypertension, diabetes, overweight or obesity, high low-density lipoprotein cholesterol, and cigarette smoking, was increased worldwide [1]. For instance, globally, 5.02 million death and 160 million disability-adjusted life years (DALYs) were attributed to high BMI in 2019. Further, the number of deaths caused by diabetes and high blood pressure increased respectively from 2.91 million and 6.79 million in 1990 to 6.50 million and 10.8 million in 2019 [1]. Different studies conducted in the general population [13,14,15] and seafarers [16,17,18,19,20] were reported that modifiable CVD risk factors included cigarette smoking, overweight or obesity, hypertension, and diabetes, can be reduced or eliminated by modifying lifestyle behaviors. Studies found that the prevalence of CVD risk factors included overweight or obesity and cigarette smoking to be higher among seafarers when compared to the general population [21,22]. There are different reasons that seafarers might have more modifiable CVD risk factors compared to the general population. Their work is both physically and psychologically stressful, working long hours and short average sleep time, prolonged time away from their family. It has been also demonstrated that work-related stress is a main contributor to CVD risk factors [21,23].

To mitigate the risk factors and the burden of CVD onboard ships among seafarers, it is important to understand the up-to-date prevalence of modifiable risk factors. Analysis of which modifiable risk factors frequently occur simultaneously is relevant for identifying a high-risk group. Understanding how clustering of CVD risk factors is associated with socio-demographic and occupational characteristics could help for developing effective CVD prevention and control strategies.

The present study has investigated the prevalence of reported modifiable CVD risk factors and their distribution by socio-demographic and occupational characteristics among seafarers. We have also investigated the association between socio-demographic and occupational variables and reported modifiable CVD risk factor clustering among seafarers. This study is probably the first investigation involving a large representative sample of seafarers, and no study has been conducted so far on reported modifiable CVD risk factors and clustering among seafarers. The present work could therefore provide up-to-date, evidence-based information on the prevalence and clustering of modifiable CVD risk factors in seafarers.

## 2. Materials and Methods

### 2.1. Study Design and Setting

In the present study, we conducted a cross-sectional epidemiological study to assess the prevalence and clustering of reported modifiable CVD risk factors among seafarers. This study was conducted from November to December 2020 onboard ships. Currently, nearly 65,000 deep-sea merchant ships operate, carrying an average of 1.3 million seafarers worldwide [24]. The workforce onboard ship is classified into three broad categories: deck, engine, and galley/support personnel. In 2015, the number of seafarers actively employed at sea, 774,000 officers, and 873,500 ratings (non-officers) [24].

### 2.2. Study Participants and Procedures

The study participants were recruited through International Radio Medical Center (C.I.R.M.). C.I.R.M. is an Italian Telemedical Maritime Assistance Service (TMAS) Center and is the organization with the largest experience worldwide in terms of the number of patients assisted onboard ships. C.I.R.M. provides teleconsultations and medical advice to seafarers and passengers regardless of their nationality and flag of the vessels 24 h a day, seven days a week, and 365/366 days a year. C.I.R.M. has more than 5000 ship contacts. Of these, 400 ships were selected randomly from the ships’ list by applying a simple random sampling strategy. In the second step, the research team presented the study’s purpose and protocol to all captains of selected vessels to obtain permission to submit a self-reported anonymous questionnaire and requested the list of seafarers per ship. The captains of 400 ships agreed to participate in the study and provided the active seafarers’ (seafarers on duty) lists per vessel. A list of a total of 8125 seafarers with indication of their names, age and ranking was obtained in a sample of 400 ships. Inclusion criteria were seafarers over the age of 18 and signing the informed consent form. In the subsequent step, a simple random sampling method to select the potential participants from the list based on our eligibility criteria was used.

All crew members were eligible for this study because they are greater than 18 years old. According to the International Labor Organization (ILO), seafarers’ recruitment policy limits seafarers’ age, and anyone who is recruited as a seafarer must be over 18 years of age [25]. As for the data collection, the research team collaborated with the C.I.R.M. doctors and provided one-day training via videoconferencing for the telemedicine case manager and one crew member per ship on survey administration and how to measure the body weight and height of the participating crew members. Telemedicine Case Managers (TCMs) are already trained medical first responders and have experience working with seafarers’ aboard ships and TMAS doctors or other health professionals. As a result, the questionnaire was sent to telemedicine case managers via their email address by the C.I.R.M., enclosing the invitation letter and informed consent forms. The survey was then administered by trained telemedicine case managers per vessel. The invitation letter contains a brief introduction to the study purpose, procedures, declaration of participant anonymity, and voluntary participation. Besides, the participants were assured of the privacy and confidentiality of the response. The participant who chose to participate provided their signed informed consent before participation in the study.

### 2.3. Data Collection

Data were collected using a standardized and anonymous questionnaire, and the tool has four core parts. The first part of the questionnaire contains the socio-demographic information included age, gender, educational status, nationality, and marital status. The second section of the questionnaire was occupational characteristics, including rank, worksite, job duration at sea, working hours per week. The third part of the questionnaire contains a history of high blood pressure (hypertension), history of diabetes, physical measurement (weight and height), alcohol consumption, and cigarette smoking status. As for the measured high blood pressure (hypertension) was ascertained with the following questions. Has a doctor or other health professional ever told you that you have high blood pressure (hypertension)? The question has only two options, “Yes” and “No”. Among those who answered “Yes” to the above question, they were further asked, “Are you currently receiving medicine for your high blood pressure (Hypertension)?”. This question also has two choices, “Yes” and “No”. Participants who answered “yes” to the above medicine question were also asked to show any antihypertensive medication they were currently taking. Regarding self-reported hypertension (HTN), in this study, it was defined as having a past hypertensive diagnosis and currently using medication due to hypertension. As for the diabetes mellitus, the self-reported diabetic Mellitus was assessed by asking, “Have you ever been told by a doctor or other health worker that you have raised blood sugar levels or diabetes?” The question has two options “Yes” and “No” and the subjects who answered yes to the above question were further asked, “Are you currently taking medicine for high blood sugar level?” The subjects who were taking medicine for high blood sugar levels were further asked to show any drug currently they were receiving due to diabetic Mellitus. In this study, self-reported diabetic mellitus (DM) was defined as having a past diabetic mellitus diagnosis and a current diabetic mellitus treatment. The current smoking was assessed by asking, “Do you currently smoke any tobacco products?” The question has two choices “Yes,” “No”. The participants who answered “Yes” for the above question were further asked, “do you currently smoked tobacco products daily?” Again, the participants who answered the above question “Yes” further assessed how many years they smoked cigarettes without stopping.

In this study, the current smoking was defined as the participants who smoked cigarettes regularly for a year and had not stopped smoking tobacco products at least six months. According to the World Health Organization (WHO) guideline [26], the study subjects’ body weight and height were measured. We then calculated body mass index as weight in kilograms (kg) divided by height in meter (m) squared (Weight (kg)/height (m)^2^). BMI was also classified into underweight (<18.5 kg/m^2^), normal body weight (18.5–24.99 kg/m^2^), overweight (25–29.99 kg/m^2^) and obesity (≥30 kg/m^2^). The questionnaire was designed for the Google survey tool (Google Forms) and the link shared to the C.I.R.M.

### 2.4. Statistical Analysis

Descriptive statistics included frequency and percentages, were determined for categorical variables to understand the distribution of socio-demographic and occupational characteristics by seafarer’s rank. We used the chi-square test to determine whether the socio-demographic and occupational characteristics were distributed homogenously by rank group and to evaluate their association with the prevalence and clustering of modifiable cardiovascular disease risk factors. The independent-sample t-test was used to examine the mean differences of continuous variables between officers and non-officers. The prevalence and clustering of the modifiable CVD risk factors were determined by Socio-demographic (age, marital status, educational level, and nationality) and occupational (rank, worksite, job duration at sea, and working hours per week) characteristics. The educational level of the participants was grouped into three categories, namely, high (completed college or university), middle (completed high school and technical school), and low (secondary and lower school). Moreover, the rank was categorized into officers (captain, deck, and engine officers) and non-officer (deck crew, engine crew, galley, and others).

The multinomial logistic regression model was performed to identify independent predictors for the cardiovascular disease risk factor clustering. Socio-demographic and occupational variables with *p*-values less than 0.25 in the univariate analysis were selected and entered multinomial logistic regression model. The clustering of modifiable CVD risk factors (dependent variable) was formed from the four modifiable risk factors: hypertension, diabetic mellitus, current smoking, and overweight or obesity. The categories were: (1) no risk factors, (2) one risk factor, and (3) two or three or four risk factors. Finally, having (1) one risk factor and (≥2) two and more than two risk factors versus (0) no risk factors (reference group) were analyzed in the model. Finally, adjusted Odds Ratio (OR) and 95% of confidence interval (95%CI) were reported.

Statistical analyses were performed using R-software [27], version 4.0.2 (R Foundation for Statistical Computing, Vienna, Austria). R-package ‘*dplyr*’ was used for data manipulation [28], and R-package ‘*summarytools*’ was used for frequencies tables, cross-tabulation, and other descriptive statistics [29]. R-package ‘tidystats’ was used for *chisq.test()* function and R-package ‘nnet’ was used for running the multinomial logistic regression model [30,31]. A two-tailed *p*-value less than 0.05 was considered statistically significant.

## 3. Results

### 3.1. Socio-Demographic and Occupational Characteristics

Out of a total of 8125 seafarers aged ≥18 years on selected vessels, 4648 seafarers volunteered to participate in the survey, with a response rate of 57.2%. We then excluded 330 participants due to missing data. Hence, a total of 4318 subjects were included in the analysis. As presented in Table 1, out of 4318 participants, 44.7% and 55.3% were officers and non-officers, respectively. The mean age of the participants was 37.94 + 10.32 (range: 19–70 years). Almost all (99.4%) participants were male, and more than seventy percent were from non-EU countries. Of the 3069 participants from non-EU countries, 58.7% and 36.7% were Filipino and Indian, respectively. More than three-fifths (69.8%) of study subjects were married, and 55.55% were deck workers. The average working hours per week was 65.96 + 10.96. 35.5% and 7.9% of officers and 38.7% and 8.6% of non-officers were overweight and obese, respectively. Some 71.5% officers and 53.3% of non-officers respectively had a high and middle level of education. Besides, 45.6% of the study subjects had 10 to 20 years job duration at sea (Table 1).

### 3.2. Prevalence of Modifiable CVD Risk Factors

#### 3.2.1. Self-Reported Hypertension

The prevalence of reported hypertension was 20.8% (95% CI: 19.6% to 22.1%). 18.5% and 27.7% of officers and non-officers respectively had reported hypertension and the prevalence of reported hypertension was significantly higher among non-officers than officers (22.7% vs. 18.5%). The prevalence of reported hypertension was high in study subjects who had married, older age groups, had a lower education level, had long job duration at sea and in deck workers regardless of rank (Table 2). Irrespective of rank, the prevalence of hypertension increased with working hours per week, from 14.1% in participants working <56 h per week to 32.2% in those working >71 h per week.

#### 3.2.2. Self-Reported Diabetes

Out of the total, 366 (8.5%) participants had reported diabetic mellitus. The prevalence of reported diabetic mellitus was higher in study participants who had married (11.2% (10.0% to 12.3%)) compared with single (2.3% (1.6% to 3.3%)). The prevalence of diabetes mellitus (DM) increased significantly with age, from 0.3% in the age group 19–30 years to 25.9% in the age ≥51 years. This risk factor’s prevalence was higher among the participants who had 21 years and above job duration at sea, working 71 h and more per week. The proportion of DM was increased with the decreasing level of education regardless of rank. As for the worksites, the engine workers had a high prevalence of DM irrespective of rank. Variables included rank and nationality, were not significantly associated with the prevalence of self-reported DM (Table 2).

#### 3.2.3. Self-Reported Current Smoking

Reported current smoking was observed in 32.5% (95% CI: 31.2% to 33.9%) of the total participants. As shown in Table 2, age groups, nationality, educational level, rank, and working hours per week were significantly associated with the prevalence of reported current smoking. In contrast, variables included marital status, worksites, and job duration at sea were not associated with current smoking. Current smoking varied significantly with age groups and was higher in non-officers than officers (34.5% vs. 30.2%). The prevalence of reported current smoking was increased with working hours per week, from 25.7% in participants who had worked ≤56 h to 40.9% in those working 71 h or more per week.

#### 3.2.4. Overweight or Obesity

Some 44.7% (95% CI: 43.2% to 46.2%) of the total participants were overweight or obese. As presented in Table 2, except nationality all socio-demographic and occupational variables were significantly associated with prevalence of the overweight or obese. The prevalence of the overweight or obese was increased with the increasing the age, working hours per week and job duration at sea regardless of rank. In contrast, this risk factor was increased with the decreasing levels of education. It was observed relatively high in non-officers compared to officers (47.3% vs. 41.4%). Besides, the prevalence of overweight or obese was found to be high in participants who had married than single (49.9% vs. 33.7%) (Table 2).

As shown in Table 3, the prevalence of CVD risk factors, except self-reported current smoking increased with age both in officers and non-officers. Reported hypertension and diabetes prevalence increased with the decreasing the level of education in non-officers, but not in officers.

Among non-officers, the prevalence of modifiable CVD risk factors increased with working hours per week. In contrast, CVD risk factors prevalence, except reported hypertension varied significantly with working hours per week among officers. The proportion of overweigh or obesity and reported diabetes increased with the length of work at sea both in officers and non-officers. Educational levels and nationality were not associated with reported hypertension and diabetes among officers. In addition, working hours per week was not associated with hypertension in officers (Table 3).

### 3.3. Clustering of Modifiable CVD Risk Factors

The clustering of modifiable CVD risk factors was categorized into three classes. The classes were no risk factors, having one risk factor and clustering two and more than two risk factors (Table 4). In general, 68.5% (95% CI: 67.2% to 69.9%) and 28.3% (26.9% to 29.7%) of the study subjects respectively, had at least one and at least two modifiable CVD risk factors. Overall, 31.4 %(30.0% to 32.8%), 40.3 (38.8% to 41.8%), 20.9 % (19.7% to 22.2%), 6% (5.4% to 6.8%) and 1.3% (1.0% to 1.7%) of the study participants respectively had zero, one, two, three and four modifiable CVD risk factors. As for the rank, 33.4 % of officers and 29.8% of non-officers had no CVD risk factors.

In contrast, 43.5% and 23.1% of officers and 37.7% and 32.5% of non-officers had one and at least two modifiable risk factors, respectively. The prevalence of having at least two risk factors increased significantly among non-officers compared with officers. The prevalence of having at least two risk factors increased significantly with age, from 13.3% in the age group 19–30 years to 51.3% in age ≥51 years. On the other hand, the prevalence of at least two risk factors of CVD increasing with the decreasing the level of education. However, the proportion of having at least two risk factors significantly increased with increasing the job duration at sea and working hours per week regardless of rank (Table 3).

As for the combination of the CVD risk factors, self-reported current smoking/overweight or obesity (30.9% (28.9% to 32.9%)), overweight or obesity/self-reported hypertension (27.5% (25.6% to 29.5%)), and self-reported current smoking/self-reported hypertension (17.5% (15.9% to 19.2%)) were the three most common of all two modifiable CVD risk factor clustering. Besides, the combination of overweight or obesity/self-reported hypertension/self-reported current smoking (70.0% (64.0% to 75.3%) were the most often clustering of three modifiable CVD risk factors. Among the participants with only one risk factors, 49.2% were overweight or obesity, 35.3% were current smokers, 10.9% had hypertension and 4.6% had diabetes.

The prevalence of modifiable CVD risk factor clustering by socio-demographic and occupational characteristics stratified by rank is presented in Table 5. Consequently, the prevalence of having at least two modifiable CVD risk factors was higher in older ages, in both officers and non-officers. The prevalence of having two or more CVD risk factors increased with working hours per week among non-officers, but not in officers. However, the prevalence of at least two CVD risk factors differed significantly with the working hours per week among officers. The proportion of having ≥2 risk factors increased with the working hours per week in non-officers and with the job duration at sea among officers. The clustering of two and more than two CVD risk factors found to be more favorable in both officers and non-officers who had married than single and the differences was also statistically significant.

As summarized in Table 6, multinomial logistic regression analysis showed that study subjects aged 51 years and above 3.92 times more likely to have at least two CVD risk factors compared to those aged from 19–30 years. Non-officers (OR: 1.36, 95% CI:1.09–1.70) were more likely to have at least two CVD risk factors when compared to officers. Participants from EU countries were 1.38 and 1.60 more likely to have one and at least two CVD risk factors than those from non-EU countries. Study subjects working 71 h and above per week and had 21 and above job duration at sea were more likely to have both one, and at least two risk factors compared with those who were working less than or equal to 56 h, and had less ten years work experiences, respectively. Besides, study individuals who had lower level of education were more likely to have at least two CVD risk factors when compared to high level of education. Variables included educational level (middle vs. higher), nationality, job duration at sea and working hours per week were independent predictors for both having one and at least two modifiable CVD risk factors. On the other hand, age, marital status, and rank were important predictors for having at least two modifiable CVD risk factors (Table 6).

## 4. Discussion

This cross-sectional epidemiological study has assessed the prevalence and clustering of reported modifiable CVD risk factors among seafarers. This study is the first study to evaluate the prevalence and clustering of reported modifiable CVD risk factors among seafarers with a large representative sample. As a result, the prevalence of reported hypertension, diabetes, current smoking, and overweight or obesity was 20.8%, 8.5%, 32.5%, and 44.7%, respectively. The most important modifiable CVD risk factor in both officers and non-officers was overweight or obesity. Compared with the previous studies [16,20,21], the prevalence of hypertension among seafarers was less in our study. There are several reasons why the prevalence of hypertension might have less in our study than in previous studies among seafarers. First, we evaluated self-reported hypertension and did not consider participants who were not taking antihypertensive treatment, although they have high blood pressure levels. These could be the reasons that underestimate the proportion of this risk factor in the present study. However, our finding was almost in line with the study conducted among Iranian seafarers and greater than the study conducted on Italian flag vessels regardless of the difference in methods [17,18].

Regardless of the difference in methods, we found a higher prevalence of both self-reported current smoking and diabetes than the previous studies carried out in seafarers [19,21]. This study documented that 36.4% and 8.3% of the participants were overweight and obese, respectively. Our result was inconsistent with studies conducted among Danish seafarers [32,33], which reported 70.8% and 76.6% overweight and 30.9% obesity. The differences might be due to differences in the methods and data sources. As for the rank, the prevalence of self-reported modifiable CVD risk factors, except diabetes was significantly higher among non-officers compared with officers. This might be due to work-related stress because, as different studies reported that non-officers work is characterized by long working hours, night shift work, short average sleep time, suffer from frequent sleep interruption, irregular working times, and more physically demanding [9,17,34,35].

Life on board is another environmental stressor for seafarers, especially for non-officers because non-officers stay on board for more extended periods than officers (8.3 months vs. 4.8 months) [35]. Due to night shift work, lack of sleep, and intense activity, seafarers, especially non-officers, experiencing various coping strategies, including smoking cigarettes, and drinking alcohol during at work. Hence, these physical and Psychosocial stressors and high levels of work-related fatigue, lack of leisure time, and physical inactivity lead to high BMI and other modifiable CVD risk factors. Besides, Work-related stressors can affect the body by activating the neuroendocrine stress pathway, and unhealthy individual lifestyle behaviors (unhealthy diet, smoking, heavy alcohol consumption, and physical inactivity) can indirectly affect the body. Several studies in general population [36,37,38,39,40] and seafarers [34,41,42,43] have reported that work-related stress contributes significantly to modifiable CVD risk factors. A study in the general population reported that work-related stress, characterized by the effort-reward imbalance model, was significantly associated with a high BMI [44]. In another general population study, also work-related stress, described by the effort-reward imbalance model, was associated with metabolic syndromes [45].

The present study reports that more than four in six (68.5%) seafarers aged 19 to 70 have at least one of the following modifiable CVD risk factors: reported hypertension, diabetes, current smoking, and overweight or obesity. Besides, the clustering of two or more two CVD risk factors was noted in 28.5% of study participants. We found that significantly higher prevalence of two or more CVD risk factors among non-officers compared with officers. It is suggested that the non-officer work accompanied with the exposure to different work-related stressors may have unfavorable effects on cardiovascular health conditions in non-officers. Our finding was inconsistent with the study conducted on German-flagged ships, which reported a higher prevalence of coronary heart disease (CHD) risk factor clustering (>3 risk factors) among officers than non-officers (crew ranks) [16]. These differences could be due to differences in methods and CVD risk factor profiles in the study. For example, we did not consider biochemical parameters such as LDL cholesterol, HDL cholesterol, and triglycerides in the present study. Our study documented that the clustering of reported current smoking/overweight or obesity and overweight or obesity/reported hypertension/current smoking was the most among the combination of two and three modifiable CVD risk factors.

We found that 33.4%, 43.5%, and 23.1% of officers and 29.8%, 37.7%, and 32.5% of non-officers respectively had zero, one, and two and more than two CVD risk factors. Another study conducted among seafarers reported that clustering of more than three CHD risk factors was observed in 56.2% of the galley staff, 43.6% of the engine officers, 32.2% of the deck officers, 24.6% of the deck crew, and 17.0% of the engine crew [16]. In the present study, modifiable CVD risk factors were observed more often in study subjects from EU countries with a prevalence between 9.2% and 45.8%. The significantly higher prevalence of both one and at least two modifiable CVD risk factors in participants from EU- countries compared to non-EU-countries mighty be due to their older age. Participants from EU countries were relatively older than those from non-EU countries. Besides, multinomial logistic regression analysis reported that participants from EU-countries 1.60 times more likely to have two and more than two CVD risk factors than those from non-EU countries. Our result was consistent with the study carried out among seafarers regardless of the differences in method, which revealed that European seafarers were 2.4 times more likely to have more than three CHD risk factors than non-European seafarers [16]. Another study reported that the proportion of high blood sugar (30%) was observed in the Croatian sailor [46].

Non-officers in the older age strata (i.e., age of 41 to 50, and age of >51) exhibited a higher prevalence of two and more CVD risk factors compared with the officers. Participants aged 51 and older were approximately four times more likely to have at least two CVD risk factors (OR: 3.92 (95% CI: 2.44–6.29)] than those aged between 19 and 30 years old, while controlling for marital status, rank, educational level, nationality, length of work at sea and working hours per week. This could be due to work-related stressors causing a negative effect on cardiovascular health after a long latency. Older workers may face more work-related stressors, are inactive in physical activity, and more able to complain about the psychological demands of work than younger age groups. Besides, older age is associated with an increased risk of various pathological changes, making older workers more exposed to different physical and psychological stressors than younger workers. A study in seafarers reported increased health problems and fatigue in older workers [47]. The same study found that the interaction between job demands, and age significantly impacted overall mental health and perceived stress [47]. Our study showed that non-officers (OR: 1.36 (95% CI: 1.09–1.70)) were more likely than the officers to have two and more than two modifiable CVD risk factors, while controlling for marital status, age, educational level, nationality, job duration at sea and working hours per week. Our result was not in line with the other previous study carried out in seafarers regardless of the difference in methods [16,48].

In general, this study revealed that non-officers, older age, married, low level of education, working hours per week, EU nationality, and job duration at sea were positively associated with modifiable CVD risk factor clustering, compared to their counterparts. Hence, the present findings may help to develop modifiable CVD risk factors prevention and control strategies onboard ships. As for intervention, for example, non-officers, those who are married, older seafarers (≥51 years), who work long hours per week, those with a low level of education, who have a long duration of work at sea, and seafarers from EU- countries could be screened for modifiable CVD risk factors, in particular for clustering of CVD risk factors. Additionally, these groups should be targeted for early prevention programs to reduce risk factors and prevent cardiovascular diseases, as they are more likely to have a modifiable CVD risk factor clustering.

Telemedicine approach prevention program can be more appropriate for screening the high-risk groups and follow-up visits at sea because Seafarers are hundreds of miles away from the nearest aid point regarding healthcare. Studies have reported that telemedicine has proven effective by providing advice, diagnosis, and treatment to seafarers during emergencies at sea [49]. Besides, it is possible to follow up visits and regular medical examinations onboard ships through telemedicine using new technologies, such as high-speed internet and video conferencing [50,51]. Promoting a healthy lifestyle or behavioral modification efforts is most important to reducing CVD risk factors. Hence, the International Maritime Organization (IMO), together with other responsible bodies and stakeholders, should take into account strategies related to cigarette smoking reduction, including smoking restrictions on board ships. In 2006, the International Labor Organization (ILO) [25] adopted the Maritime Labor Convention (MLC) 2006, and it entered into force on 20 August 2013. In chapter five (title two), the 2006 Convention deals with the timetable working hours and rest hours for seafarers and clearly stated that seafarers’ working hours are eight per day with one day of rest per week. However, our study reported that 51.2% and 22.9% of the participants worked 57–70 h and 71+ h per week, respectively. Therefore, the Convention 2006 has not yet been fully applied related to working hours of seafarers on board ships. Hence, the ILO and other responsible bodies should pay attention to its enforcement related to working hours according to the guideline because long working hours per week was one of the independent predictors for modifiable CVD risk factor clustering. As for physical activity, the working conditions of seafarers does not motivate them to carry out daily physical training as on land because there are sudden climate changes, accidents, and physical and psychological stress. However, planned health education regarding physical activity through telemedicine and the provision of simple and easy-to-use mobile applications [52] could encourage the seafarers to practice daily physical exercises to be physically active and reduce the chance of being overweight or obese.

Limitation of this study: this study was a cross-sectional study, and the study design prevents us from determining the causality or temporal relationship between modifiable CVD risk factor clustering and CVD. In the present study, we assessed self-reported modifiable CVD risk factors, except overweight or obesity. We did not include participants who were not on treatment despite having high blood pressure or high blood sugar. Therefore, these could underestimate the prevalence of hypertension or diabetes. Current smoking may be subject to reporting bias as it depends only on the participants’ responses. Besides, those who did not smoke regularly were excluded. Hence, the prevalence of current smoking may be underestimated.

## 5. Conclusions

The results of our study demonstrate that a high prevalence of reported modifiable CVD risk factors was observed in non-officers. This study reported that the clustering of reported current smoking/overweight or obesity and overweight or obesity/reported hypertension/ reported current smoking were the most among the combination of two and three modifiable CVD risk factors; the prevalence was 30.9% and 70.0%, respectively. The present study indicates that non-officers, older workers, European seafarers, participants with a low level of education, who work long hours a week, are married, and have a long job duration at sea are susceptible to modifiable CVD risk factor clustering. Hence, a specific intervention targeting the high-risk groups should be designed and implemented onboard ships. In the present study, we did not consider cholesterol profiles, so a prospective study should be considered in the future to evaluate clustering and predict modifiable CVD risk factors, including cholesterol profiles among seafarers, for further preventive measures.

## Figures and Tables

**Table 1:** Distribution of socio-demographic, occupational, and other relevant characteristics of participants by the rank group.

Variables	Total (%)	Rank		*p*-Value
		Officer, n (%)	Non-Officer n (%)	
Number, n (%)	4318 (100.0)	1929 (44.7)	2389 (55.3)	
Age (in years), mean (SD)	37.94 ± 10.32	38.39 ± 9.89	37.58 ± 10.60	0.011
Gender (male)	4290 (99.4)	1925 (99.8)	2365 (98.9)	-
Nationality				<0.001
EU-countries	1222 (28.3)	782 (40.5)	440 (18.4)	
Non-EU countries	3096 (71.7)	1147 (59.5)	1949 (81.6)	
Marital status				0.001
Single	1303 (30.2)	526 (27.3)	777 (32.5)	
Married	3015 (69.8)	1403 (72.7)	1612 (67.5)	
Educational level				<0.001
Higher	1741 (40.3)	1375 (71.3)	366 (15.3)	
Middle	1803 (41.7)	530 (27.5)	1273 (53.3)	
Low	774 (18)	24 (1.2)	750 (31.4)	
Worksite				<0.001
Deck	2396 (55.5)	1167 (60.5)	1229 (51.4)	
Engine	1468 (34)	762 (39.5)	706 (29.6)	
Galley	454 (10.5)	0 (0)	454 (19)	
Job duration at sea				<0.001
<10 years	1551 (35.9)	481 (24.9)	1070 (44.8)	
10–20 years	1967 (45.6)	1000 (40.4)	967 (40.5)	
21+ years	800 (18.5)	448 (14.7)	352 (14.7)	
Working hours per week, mean (SD)	65.96 ± 10.98	65.67 ± 10.4	66.19 ± 11.4	0.122
Body mass index (BMI)				<0.001
Underweight	34 (0.8%)	8 (0.4%)	26 (1.0%)	
Normal	2355 (54.5%)	1123 (58.2%)	1232 (51.6%)	
Overweight	1571 (36.4%)	646 (33.5%)	925 (38.7%)	
Obesity	358 (8.3%)	152 (7.9%)	206 (8.6%)	

**Table 2:** Prevalence (95% CI) of modifiable risk factors of cardiovascular disease and its distribution by socio-demographic and occupational characteristics among seafarers.

	Self-Reported Hypertension	Self-Reported Diabetes	Self-Reported Current Smoking	Overweight or Obesity
**Total**	20.8 (19.6–22.1)	8.5 (7.7–9.4)	32.5 (31.2–33.9)	44.7 (43.2–46.2)
Age group (in years)				
19–30	3.6 (2.7–4.9)	0.3 (0.10–0.89)	36.3 (33.6–39.0)	33.8 (31.2–36.6)
31–40	16.6 (14.7–18.7)	5.2 (4.3–6.9)	32.9 (30.5–35.5)	43.5 (40.9–46.2)
41–50	35 (32.2–37.8)	13.4 (11.5–15.5)	25.7 (23.2–28.3)	50.4 (47.4–53.3)
51+	41.6 (37.3–47.9)	25.9 (22.3–29.9)	37.9 (33.7–42.3)	60.9 (56.6–65.2)
*p*-value	<0.001	<0.001	<0.001	<0.001
Nationality				
EU-countries	22.6 (20.3–25.0)	9.2 (7.7–11.0)	41.5 (38.7–44.3)	45.8 (43–48.6)
Non-EU countries	20.2 (18.8–21.6)	8.2 (7.2–9.2)	29.2 (27.4–30.6)	44.2 (42.5–45.9)
*p*-value	0.084	0.279	<0.001	0.356
Marital status				
Single	9.8 (8.2–11.5)	2.3 (1.6–3.3)	34.3 (31.7–36.9)	33.7 (31.2–36.3)
Married	25.6 (24.1–27.2)	11.2 (10–12.3)	31.8 (30.2–33.5)	49.9 (47.6–51.2)
*p*-value	<0.001	<0.001	0.111	<0.001
Educational level				
Higher	15.2 (13.4–16.8)	7.4 (6.2–8.8)	28.7 (26.6–30.9)	37.5 (35.2–39.8)
Middle	23.7 (21.8–25.8)	8.7 (7.4–10.0)	36.7 (34.4–38.9)	47.1 (44.8–49.4)
Low	27.2 (24–30.4)	10.5 (8.4–12.9)	31.5 (28.3–34.9)	55.3 (51.7–58.8)
*p*-value	<0.001	0.037	<0.001	<0.001
Rank				
Officer	18.5 (16.8–20.3)	7.7 (6.5–8.9)	30.2 (28.2–32.3)	41.4 (39.2–43.6)
Non-officer	22.7 (21–24.5)	9.2 (8.0–10.4)	34.5 (32.5–36.4)	47.3 (45.3–49.4)
*p*-value	<0.001	0.099	0.003	<0.001
Worksite				
Deck	23.5 (21.8–25.2)	8.1 (7.1–9.3)	33.5 (31.6–34.4)	43.7 (41.7–45.7)
Engine	18.5 (16.6–20.6)	9.7 (8.3–11.4)	31.5 (29.1–33.9)	44.5 (41.9–47.1)
Galley	14.5 (11.5–18.2)	6.2 (4.2–8.9)	31 (26.9–35.6)	50.2 (45.6–54.9)
*p*-value	<0.001	0.038	0.338	0.038
Job duration at sea				
<10 years	6.6 (5.5–8.0)	1.4 (0.9–2.1)	34.4 (32.2–36.8)	32.2 (29.7–34.5)
10–20 years	26.5 (24.6–28.5)	9.9 (8.7–11.4)	31.6 (29.6–33.7)	49.5 (47.2–51.7)
21+ years	34.5 (31.2–37.9)	18.6 (16–21.5)	31.3 (28.1–34.6)	57.4 (53.9–60.8)
*p*-value	<0.001	<0.001	0.156	<0.001
Working hours per week				
≤56 h				
57–70 h	14.1 (12.2–16.3)	5.4 (4.1–6.9))	25.7 (23.2–28.4)	38.0 (35.2–40.9)
71+ h	19.2 (17.6–20.9)	9.1 (7.9–10.4))	32.2 (30.3–34.2)	46.5 (44.4–48.6)
*p*-value	32.2 (29.3–35.2)	10.6 (8.8–12.8)	40.9 (37.9–44.2)	48.3 (45–51.5)
	<0.001	<0.001	<0.001	<0.001

**Table 3:** Prevalence (95% CI) of modifiable CVD risk factors by socio-demographic and occupational characteristics stratified by rank.

Rank Group	Self-Reported Hypertension	Self-Reported Diabetes	Self-Reported Current Smoking	Overweight or Obesity
**Officer**				
**Overall**	18.5 (16.8–20.3)	7.7 (6.5–8.9)	30.2 (28.1–32.3)	41.4 (39.2–43.6)
Age group (in years)				
19–30	5.1 (3.4–7.6)	0.2 (0.01–1.4)	36.2 (31.9–40.7)	30.9 (26.7–35.3)
31–40	14.8 (12.3–17.6)	1.5 (0.8–2.8)	32.7 (29.3–36.3)	39.0 (35.4–42.7)
41–50	26.3 (22.6–30.4)	15.4 (12.4–18.9)	18.5 (15.3–22.2)	43.5 (39.2–47.9)
51+	40.4 (33.9–47.1)	25.0 (19.6–31.2)	36.0 (29.8–42.6)	65.8 (59.2–71.8)
*p*-value	<0.001	<0.001	<0.001	<0.001
Nationality				
EU-countries	19.7 (16.9–22.7)	7.9 (6.2–10.1)	38.9 (35.5–42.4)	44.1 (40.6–47.7)
Non-EU countries	17.7 (15.6–20.1)	7.5 (6.1–9.2)	24.2 (21.8–26.8)	39.5 (36.7–42.4)
*p*-value	0.295	0.794	<0.001	0.058
Marital status				
Single	12.9 (10.2–16.2)	4.0 (2.6–6.1)	33.7 (29.7–37.9)	28.9 (25.1–33.0)
Married	20.6 (18.5–22.8)	9.1 (7.6–10.7)	28.9 (26.5–31.3)	46.0 (43.4–48.7)
*p*-value	<0.001	<0.001	0.047	<0.001
Educational level				
Higher	17.5 (15.6–19.7)	8.4 (6.9–9.9)	28.2 (25.9–30.7)	37.6 (35.0–40.2)
Middle	21.5 (18.1–25.3)	6.2 (4.4–8.7)	33.0 (29.1–37.2)	49.2 (44.9–53.6)
Low	8.3 (1.5–2.8)	0.0	79.2 (57.2–92.1)	83.3 (61.8–94.5)
*p*-value	0.058	0.106	<0.001	<0.001
Worksite				
Deck	18.8 (16.6–21.2)	6.2 (4.9–7.7)	31.1 (28.5–33.9)	42.9 (40.1–45.8)
Engine	18.1 (15.1–21.1)	10.0 (7.9–12.4)	28.7 (25.6–32.1)	39.0 (35.5–42.5)
Galley	__	__	__	__
*p*-value	N/A	N/A	N/A	N/A
Job duration at sea				
<10 years	6.0 (4.1–8.6)	0.4 (0.07–1.7)	33.5 (29.3–37.9)	26.0 (22.2–30.2)
10–20 years	16.6 (14.4–19.1)	5.8 (4.5–7.5)	30.8 (27.9–33.9)	41.6 (38.5–44.7)
21+ years	36.2 (31.7–40.8)	19.6 (16.1–23.7)	25.2 (21.3–29.6)	57.4 (52.6–61.9)
*p*-value	<0.001	<0.001	0.019	<0.001
Working hours per week				
≤56 h	21.3 (17.9–25.2)	5.7 (3.9–8.2)	25.4 (21.7–29.5)	37.9 (33.7–42.3)
57–70 h	16.8 (14.6–19.3)	10.6 (8.8–12.7)	32.5 (29.6–35.5)	45.2 (42.1–48.3)
71+ h	19.1 (15.5–23.3)	2.9 (1.6–5.2)	30.3 (25.9–34.9)	36.3 (31.7–41.2)
*p*-value	0.102	<0.001	0.018	0.002
**Non-officer**				
**Overall**	22.7 (21.1–24.5)	9.2 (8.0–10.4)	34.5 (32.5–36.4)	47.3 (45.3–49.4)
Age group (in years)				
19–30	2.7 (1.7–4.2)	1.8 (1.0–3.1)	36.3 (32.9–39.9)	35.7 (32.3–39.2)
31–40	18.6 (15.8–21.7)	9.2 (7.2–11.6)	33.1 (29.7–36.8)	48.3 (44.5–52.1)
41–50	41.9 (38.1–45.8)	11.1 (8.9–13.9)	31.4 (27.8–35.1)	55.8 (51.9–59.7)
51+	42.6 (36.8–48.5)	23.9 (19.2–29.3)	39.4 (33.8–45.4)	57.1 (51.2–62.8)
*p*-value	<0.001	<0.001	<0.055	<0.001
Nationality				
EU-countries	27.7 (23.6–32.2)	9.3 (6.8–12.5)	46.1 (41.4–50.9)	48.9 (44.1–53.6)
Non-EU countries	21.6 (19.8–23.5)	9.0 (7.9–10.5)	31.8 (29.7–33.9)	47.0 (44.8–49.2)
*p*-value	0.007	0.949	<0.001	0.513
Marital status				
Single	7.6 (5.9–9.7)	2.2 (1.3–3.6)	34.7 (31.4–38.2)	36.9 (33.6–40.5)
Married	30.0 (27.8–32.3)	12.5 (10.9–14.5)	34.3 (31.9–36.7)	52.4 (49.9–54.8)
*p*-value	<0.001	<0.001	0.343	<0.001
Educational level				
Higher	5.7 (3.7–8.8)	3.8 (2.2–6.5)	30.6 (25.9–35.6)	36.9 (31.9–42.1)
Middle	24.7 (22.3–27.1)	9.7 (8.1–11.5)	38.2 (35.5–40.9)	46.2 (43.4–48.9)
Low	27.7 (24.6–31.1)	10.8 (8.7–13.3)	30.0 (26.8–33.4)	54.4 (50.7–57.9)
*p*-value	<0.001	<0.001	<0.001	<0.001
Worksite				
Deck	27.9 (25.4–30.5)	10.0 (8.4–11.9)	35.7 (33.0–38.5)	44.5 (41.7–47.3)
Engine	19.0 (16.2–22.1)	9.5 (7.5–11.9)	34.4 (30.9–38.1)	50.4 (46.7–54.2)
Galley	14.5 (11.5–18.2)	6.2 (4.2–8.9)	31.1 (26.9–35.6)	50.2 (45.5–54.9)
*p*-value	<0.001	0.048	0.203	0.015
Job duration at sea				
<10 years	6.9 (5.5–8.6)	1.8 (1.1–2.8)	34.8 (31.9–37.7)	34.8 (31.9–37.7)
10–20 years	36.7 (33.7–39.8)	14.3 (12.1–16.7)	32.5 (29.5–35.5)	57.4 (54.4–60.7)
21+ years	32.4 (27.6–37.6)	17.3 (13.6–21.8)	38.9 (33.8–44.3)	57.6 (52.0–62.6)
*p*-value	<0.001	<0.001	0.089	<0.001
Working hours per week				
≤56 h	8.1 (6.2–10.7)	5.0 (3.5–7.2)	25.9 (22.5–29.6)	38.1 (34.3–42.1)
57–70 h	21.2 (18.9–23.6)	7.8 (6.4–9.5)	32.0 (29.4–34.7)	47.5 (44.6–50.4)
71+ h	41.5 (37.5–45.7)	16.2 (13.3–19.5)	48.7 (44.5–52.9)	56.9 (52.7–60.9)
*p*-value	<0.001	<0.001	<0.001	<0.001

**Table 4:** Prevalence (95% CI) of modifiable CVD risk factor clustering based on socio-demographic and occupational characteristics among seafarers.

Category	None	One	Clustering (≥2)	*p*-Value
**Total**	31.4 (30.0–32.8)	40.3 (38.8–41.8)	28.3 (26.9–29.7)	
Age group (in years)				<0.001
19–30	39.3 (36.5–42.1)	47.5 (44.6–50.2)	13.3 (11.5–15.3)	
31–40	33.5 (31.0–36.0)	41.3 (38.7–43.9)	25.2 (22.9–27.5)	
41–50	28.9 (26.4–31.7)	33.2 (30.5–35.9)	37.8 (35.0–40.7)	
51+	12.6 (9.9–15.8)	36.2 (32.1–40.5)	51.3 (46.9–55.6)	
Nationality				<0.001
EU-countries	24.9 (22.5–27.4)	42.8 (40–45.6)	32.3 (29.7–35.0)	
Non-EU countries	34.0 (32.4–35.7)	39.3 (37.6–41)	26.7 (25.2–28.3)	
Marital status				<0.001
Single	39.9 (37.3–42.7)	45.2 (42.3–47.8)	14.9 (13.0–17.0)	
Married	27.7 (26.2–29.4)	38.2 (36.5–39.9)	34.1 (32.4–35.8)	
Educational level				<0.001
Higher	38.5 (36.2–40.8)	41.6 (39.3–43.9)	19.9 (18.1–21.9)	
Middle	26.2 (24.2–28.3)	42.2 (39.9–44.5)	31.7 (29.5–33.9)	
Low	27.8 (24.7–31.0)	32.9 (29.7–34.4)	39.3 (35.8–42.8)	
Rank				<0.001
Officer	33.4 (31.3–35.6)	43.5 (41.3–45.7)	23.1 (22.3–25.1)	
Non-officer	29.8 (28.0–31.7)	37.7 (35.7–39.7)	32.5 (30.6–34.4)	
Worksite				<0.001
Deck	31.9 (30.0–33.8)	37.8 (35.8–39.7)	30.3 (28.5–32.2)	
Engine	29.2 (26.9–31.6)	45.8 (43.3–48.4)	25.0 (22.8–27.3)	
Galley	36.3 (31.9–40.9)	35.5 (30.0–40.0)	28.2 (24.1–32.6)	
Job duration at sea				<0.001
<10 years	42.8 (40.4–45.3)	42.0 (39.6–44.5)	15.2 (13.4–17.1)	
10–20 years	26.8 (24.9–28.8)	40.0 (37.8–42.2)	33.2 (31.1–35.3)	
21+ years	20.8 (18.0–23.8)	37.5 (34.2–40.9)	41.8 (38.3–45.3)	
Working hours per week				<0.001
≤56 h	43.7 (40.8–46.7)	36.0 (33.2–38.9)	20.3 (17.9–22.7)	
57–70 h	28.5 (26.6–30.4)	43.2 (41.1–45.3)	28.3 (26.5–30.3)	
71+ h	24.0 (21.5–26.9)	38.6 (35.5–41.7)	37.3 (34.3–40.5)	

**Table 5:** Prevalence (95% CI) of modifiable CVD risk factor clustering among seafarers stratified by rank.

Rank Group	None	One	Clustering (≥2)	*p*-Value
**Officer**				
**Overall**	33.4 (31.3–35.6)	43.5 (41.3–45.7)	23.1 (22.3–25.1)	
Age group (in years)				<0.001
19–30	39.8 (35.4–44.4)	48.3 (43.7–52.9)	11.9 (9.2–15.3)	
31–40	37.6 (34.1–42.3)	42.2 (38.6–45.9)	20.2 (17.4–23.4)	
41–50	30.8 (26.9–35.0)	43.7 (39.3–48.1)	25.5 (21.8–29.6)	
51+	12.7 (8.8–17.9)	37.3 (31.0–43.9)	50.0 (43.6–56.4)	
Nationality				<0.001
EU-countries	28.9 (25.8–32.2)	42.2 (38.7–45.8)	28.9 (25.8–32.2)	
Non-EU countries	36.4 (33.6–39.3)	44.4 (41.5–47.3)	19.2 (16.9–21.6)	
Marital status				<0.001
Single	44.3 (40.0–48.7)	41.4 (37.2–45.8)	14.3 (11.4–17.6)	
Married	29.3 (26.9–31.8)	44.3 (41.6–46.9)	26.4 (24.2–28.8)	
Educational level				<0.001
Higher	36.6 (34.0–39.2)	43.2 (40.6–45.9)	20.2 (18.1–22.5)	
Middle	26.2 (22.6–30.2)	45.5 (41.2–49.8)	28.3 (24.5–32.4)	
Low	8.3 (1.5–28.5)	16.7 (5.5–38.2)	75.0 (52.9–89.4)	
Worksite				N/A
Deck	32.8 (30.1–35.6)	43.2 (40.3–46.1)	24.0 (21.6–26.6)	
Engine	34.3 (30.9–37.8)	44.0 (40.4–47.6)	21.8 (18.9–24.9)	
Galley	___	____	___	
Job duration at sea				<0.001
<10 years	47.6 (43.1–52.2)	40.7 (36.3–45.3)	11.6 (8.9–14.9)	
10–20 years	32.8 (29.9–35.8)	45.9 (42.8–49.0)	21.3 (18.8–23.9)	
21+ years	19.4 (15.9–23.5)	41.1 (36.5–45.8)	39.5 (34.9–44.2)	
Working hours per week				<0.001
≤56 h	40.2 (35.9–44.7)	37.9 (33.7–42.3)	21.9 (18.4–25.8)	
57–70 h	28.9 (26.2–31.9)	45.3 (42.5–48.7)	25.5 (22.8–28.3)	
71+ h	35.8 (31.2–40.7)	45.6 (40.4–50.2)	18.8 (15.3–23.1)	
**Non-officer**				
**Overall**	29.8 (28.0–31.7)	37.7 (35.7–39.7)	32.5 (30.6–34.4)	
Age group (in years)				<0.001
19–30	39.0 (35.5–42.5)	46.9 (43.4–50.5)	14.1 (11.8–16.8)	
31–40	29.2 (25.9–32.8)	40.4 (36.7–44.2)	30.4 (26.9–33.9)	
41–50	27.5 (24.1–31.2)	24.9 (21.6–28.4)	47.6 (43.7–51.5)	
51+	12.5 (8.9–16.9)	35.3 (29.8–41.1)	52.2 (46.3–58.1)	
Nationality				<0.001
EU-countries	17.7 (14.3–21.7)	43.9 (39.2–48.6)	38.4 (33.9–43.2)	
Non-EU countries	32.6 (30.5–34.7)	36.3 (34.1–38.5)	31.1 (29.1–33.3)	
Marital status				<0.001
Single	37.1 (33.7–40.6)	47.5 (43.9–51.1)	15.4 (13.1–18.2)	
Married	26.4 (24.2–28.6)	32.9 (30.7–35.3)	40.7 (38.3–43.1)	
Educational level				<0.001
Higher	45.6 (40.5–50.9)	35.5 (30.7–40.7)	18.9 (15.1–23.3)	
Middle	26.2 (23.8–28.7)	40.8 (38.1–43.5)	33.1 (30.5–35.7)	
Low	28.4 (25.2–31.8)	33.5 (30.1–36.9)	38.1 (34.7–41.7)	
Worksite				<0.001
Deck	31.0 (28.4–33.7)	32.6 (30.0–35.3)	36.4 (33.7–39.1)	
Engine	23.7 (20.6–27.0)	47.9 (44.0–51.6)	28.5 (25.2–31.9)	
Galley	36.3 (31.9–40.9)	35.5 (31.1–40.1)	28.2 (24.1–32.6)	
Job duration at sea				<0.001
<10 years	40.7 (37.7–43.7)	42.6 (39.6–45.6)	16.7 (14.5–19.1)	
10–20 years	20.6 (18.1–23.3)	33.0 (30.9–37.0)	45.5 (42.3–48.7)	
21+ years	22.4 (18.3–27.2)	33.9 (28.1–38.2)	44.6 (39.4–49.9)	
Working hours per week				<0.001
≤56 h	46.6 (42.6–50.6)	34.5 (30.8–38.5)	18.9 (15.9–22.3)	
57–70 h	28.1 (25.6–30.7)	41.2 (38.4–44.0)	30.8 (28.2–33.5)	
71+ h	15.7 (12.8–18.9)	33.7 (29.9–37.8)	50.6 (46.5–54.8)	

**Table 6:** Multinomial logistic regression analysis of modifiable cardiovascular disease risk factor clustering among seafarers (n = 4318).

Category	One		Clustering (≥2)	
	OR (95% CI)	*p*-Value	OR (95% CI)	*p*-Value
Age group (in years)				
19–30	1	-	1	-
31–40	0.85 (0.78–1.10)	0.219	1.02 (0.74–1.39)	0.913
41–50	0.92 (0.86–1.23)	0.068	1.27 (0.89–1.83)	0.193
51+	1.04 (0.67–1.61)	0.871	3.92 (2.44–6.29)	<0.001
Marital status				
Single	1		1	
Married	1.18 (0.97–1.43)	0.114	1.59 (1.24–2.03)	<0.001
Educational level				
Higher	1		1	
Middle	1.56 (1.30–1.88)	<0.001	2.21 (1.78–2.75)	<0.001
Low	1.25 (0.97–1.62)	0.084	2.48 (1.87–3.30)	<0.001
Nationality				
Non-EU countries	1		1	
EU-countries	1.38 (1.16–1.64)	<0.001	1.60 (1.31–1.95)	<0.001
Rank				
Officer	1		1	
Non-officer	1.07 (0.88–1.31)	0.485	1.36 (1.09–1.70)	0.007
Job duration at sea				
<10 years	1		1	
10–20 years	2.22 (1.77–2.79)	<0.001	2.73 (2.09–3.57)	<0.001
21+ years	2.37 (1.68–3.35)	<0.001	2.60 (1.79–3.78)	<0.001
Working hours per week				
≤56 h	1		1	
57–70 h	1.73 (1.46–2.05)	<0.001	2.03 (1.65–2.49)	<0.001
71+ h	1.88 (1.52–2.33)	<0.001	3.08 (2.42–3.92)	<0.001

## Data Availability

The data presented in this study are available on request from the corresponding author.
